# *In vitro* and *in vivo* Synergistic Effects of Florfenicol and Thiamphenicol in Combination Against Swine *Actinobacillus pleuropneumoniae* and *Pasteurella multocida*

**DOI:** 10.3389/fmicb.2019.02430

**Published:** 2019-10-30

**Authors:** Porjai Rattanapanadda, Hung-Chih Kuo, Thomas W. Vickroy, Chi-Hsuan Sung, Tirawat Rairat, Tsai-Lu Lin, Sze-Yu Yeh, Chi-Chung Chou

**Affiliations:** ^1^Department of Veterinary Medicine, College of Veterinary Medicine, National Chung Hsing University, Taichung, Taiwan; ^2^Department of Veterinary Medicine, College of Veterinary Medicine, National Chiayi University, Chiayi, Taiwan; ^3^Department of Physiological Sciences, College of Veterinary Medicine, University of Florida, Gainesville, FL, United States

**Keywords:** synergism, florfenicol, thiamphenicol, *Actinobacillus pleuropneumoniae*, *Pasteurella multocida*

## Abstract

Potential synergism between florfenicol (FF) and thiamphenicol (TAP) was investigated for *in vitro* efficacy against *Actinobacillus pleuropneumoniae* and/or *Pasteurella multocida* as well as *in vivo* efficacy in swine. Among isolates of *A. pleuropneumoniae* (*n* = 58) and *P. multocida* (*n* = 79) from pigs in Taiwan that were tested, high percentages showed resistance to FF (52 and 53%, respectively) and TAP (57 and 53%, respectively). Checkerboard microdilution assay indicated that synergism [fractional inhibitory concentration index (FICI) ≤ 0.5] was detected in 17% of *A. pleuropneumoniae* (all serovar 1) and 24% of *P. multocida* isolates. After reconfirming the strains showing FICI ≤ 0.625 with time kill assay, the synergism increased to around 32% against both bacteria and the number could further increase to 40% against resistant *A. pleuropneumoniae* and 65% against susceptible *P. multocida* isolates. A challenge-treatment trial in pigs with *P. multocida* showed that the FF + TAP dosage at ratios correspondent to their MIC deduction was equally effective to the recommended dosages. Further on the combination, the resistant mutation frequency is very low when *A. pleuropneumoniae* is grown with FF + TAP and similar to the exposure to sub-inhibitory concentration of FF or TAP alone. The degree of minimum inhibitory concentration (MIC) reduction in FF could reach 75% (1/4 MIC) or more (up to 1/8 MIC for *P. multocida*, 1/16 for *A. pleuropneumoniae*) when combined with 1/4 MIC of TAP (or 1/8 for *A. pleuropneumoniae*). The synergism or FICI ≤ 0.625 of FF with oxytetracycline (47%), doxycycline (69%), and erythromycin (56%) was also evident, and worth further investigation for FF as a central modulator facilitating synergistic effects with these antimicrobials. Taken together, synergistic FF + TAP combination was effective against swine pulmonary isolates of *A. pleuropneumoniae* and *P. multocida* both *in vitro* and *in vivo.* Thus, this study may offer a potential alternative for the treatment of *A. pleuropneumoniae* and *P. multocida* infections and has the potential to greatly reduce drug residues and withdrawal time.

## Introduction

The continuing emergence of antimicrobial resistance coupled with the slow development of new antimicrobial drugs represents a growing worldwide challenge for both human and animal healthcare (Cheng et al., 2016). In one of the approaches to address this pressing problem, clinical research has focused on the discovery of synergistic actions by novel antimicrobial combinations (Singh and Yeh, 2017), which could be implemented without need to modify existing drugs. Benefits associated with the use of combined antimicrobial therapy with synergistic activities include the potential for delayed development of bacterial resistance, a broadening of antibacterial spectrum to treat polymicrobial infections, a reduction in drug toxicity and reduced cost or risk of harmful residues in food products (Eliopoulos and Eliopoulos, 1988; Ahmed et al., 2014). One of the best known models to measure the effects of antimicrobial drug combination is the checkerboard assay in which a two dimensional array of serial concentrations of test compounds is used as the basis for calculation of fractional inhibitory concentration index or FICI (White et al., 1996). The “Time-kill” experiment is another method that offers dynamic observation of the interaction of two antimicrobial agents over time with confirmation of the synergistic activities by checkerboard results (Grzybowska et al., 2004).

Florfenicol has been authorized for veterinary antimicrobial use in swine in many countries including European Union, United Kingdom, Japan, United States, Canada, and Taiwan (European Medicines Agency [EMA], 1999; Veterinary Medicines Directorate [VMD], 2013; The Japan Food Chemical Research Foundation [JFCRF], 2015; Bureau of Animal and Plant Health Inspection and Quarantine [BAPHIQ], 2019; The United States Food and Drug Administration [US FDA], 2019; Veterinary Drugs Directorate [VDD], 2019), while thiamphenicol is also approved in these regions except for the North America. The wide range of applications and the use as essential treatments against specific infections, in addition to the lack of sufficient therapeutic alternatives, make amphenicols [including florphenicol (FF) and thiamphenicol (TAP)] critically important antimicrobial agents for veterinary use (VCIA) according to the World Organization for Animal Health (OIE) (OIE, 2015).

Synergy between two agents within the same class of antibiotics is rarely observed *in vitro*, *in vivo* or in clinical practice. In humans, the only example of reported synergism between the same class of antibiotics is the use of dual ß lactam antibiotics. For instance, cefotaxime plus amoxicillin and ceftriaxone plus ampicillin increased activity against resistant *enterococci* compared to either in isolation (Mainardi et al., 1995; Pericas et al., 2018). In addition, unconventional use of a double carbapenem combination (meropenem + ertapenem) has revealed synergism against carbapenem-resistant *Klebsiella pneumonia* (CR-Kp) both *in vitro* and *in vivo* as well as in clinical settings (Ceccarelli et al., 2013; Oliva et al., 2015). More recently, the 2015 Infectious Diseases Society of America (IDSA) guidelines highlighted dual beta-lactam therapy as a first-line treatment option for adult infective endocarditis (Bartash and Nori, 2017). In animals, synergism within the amphenicol group was first reported recently, with FICI ≤ 0.625 detected *in vitro* against both methicillin-susceptible (3/9) and methicillin-resistant isolates (5/11) of *Staphylococcus aureus* derived from chickens, cattle and pigs as well as *in vivo* synergism in mice at half of the recommended dose of FF (10 mg/kg) plus an ineffective dose of TAP (10 mg/kg) (Wei et al., 2016a). In addition, isolates with FICI value ≤ 0.625 was detected *in vitro* for isolates of *P. multocida* (10/23) isolated from pigs, ducks and geese, *Streptococcus suis* (2/13) and *Staphylococcus hyicus* (1/6) isolated from pigs, as well as *in vivo* synergism against *P. multocida* in chickens (Wei et al., 2016b). A recent publication demonstrated *in vitro* FF and TAP with FICI ≤ 0.75 and *in vivo* efficacy at reduced dosage against *Aeromonas hydrophila* in Nile tilapia (Assane et al., 2019). While these results indicate preferential synergism by FF + TAP combination against certain bacterial species from pigs and fish and *P. multocida* from chickens, it remains unclear whether the synergistic actions extend to *P. multocida* or other major respiratory pathogens in pigs. Therefore, the purpose of the study was to determine the efficacy of a synergistic FF + TAP combination against important swine bacterial respiratory pathogens, namely *A. pleuropneumoniae* and *P. multocida* both *in vitro* and *in vivo*. Furthermore, the effectiveness of FF combinations with other antimicrobial agents were evaluated with particular emphasis on drugs that are available as injectable dosage forms.

## Materials and Methods

### Antimicrobial Agents, Bacteria, and Culture Conditions

The FF, TAP, oxytetracycline (OTC), doxycycline (DOX), erythromycin (ERY), and tylosin (TYL) were purchased from Sigma-Aldrich (St. Louis, MO, United States). All the drugs used were analytical reference standards with a purity ≥95%. Clinical isolates of *A. pleuropneumoniae* (58) and *P. multocida* (79) were obtained from the Department of Veterinary Medicine, National Chiayi University, Taiwan. All bacterial isolates were obtained originally from the lung tissue of naturally infected pigs and their species and serovars had been established beforehand using biochemical tests, PCR analysis and Multilocus Sequence Typing (MLST) (Yeh et al., 2017; Liao et al., 2019). For routine culture, *P. multocida* was grown on chocolate agar (Creative Lifescience, New Taipei city, Taiwan) and incubated at 37°C for 16–18 h, while *A. pleuropneumoniae* was also grown on chocolate agar at 37°C with 5% CO_2_ for 20–24 h.

### *In vitro* Susceptibility Testing

MIC determinations were performed according to the broth microdilution method as described in the Clinical and Laboratory Standard Institute guidelines (Clinical and Laboratory Standards Institute [CLSI], 2018) for *P. multocida*, while a modified method was used for *A. pleuropneumoniae*. The methods are briefly outlined below. For *P. multocida*, serial two-fold dilutions of FF, TAP, OTC, DOX, ERY and TYL were performed in cation-adjusted Mueller-Hinton II broth (CAMHB) (Difco Laboratories, Detroit, Michigan, United States) in a 96-well U bottom microplate. The inoculum was prepared in Brain-Heart-Infusion (BHI) broth. Bacteria at a final concentration of 5 × 10^5^ colony-forming unit (CFU)/mL were inoculated into the wells and grown at 37°C for 16–18 h. For *A. pleuropneumoniae*, serial two-fold dilutions of FF and TAP were performed in CAMHB with the addition of 15 μg/mL nicotinamide adenine dinucleotide (NAD) (Sigma-Aldrich, St. Louis, MO, United States). The inoculum was prepared in BHI supplemented with 15 μg/mL NAD (Tremblay et al., 2017; Xie et al., 2017; Ramírez-Castillo et al., 2018). Bacteria also at a final concentration of 5 × 10^5^ colony-forming unit (CFU)/mL were inoculated into the wells and grown at 37^*o*^C for 20–24 h with 5% CO_2_. The MIC was defined as the lowest concentration with no visible growth. All MIC assays in this study were at least repeated in duplicate. Four reference standard strains of bacteria, namely *Enterococcus faecalis* (ATCC 29212), *S. aureus* (ATCC 29213), *Escherichia coli* (ATCC 25922), and *A. pleuropneumoniae* (ATCC 27090) purchased from American Type Culture Collection (Manassas, VA, United States) were used as reference bacteria to verify the accuracy of all antimicrobial susceptibility tests according to the CLSI guideline (2018). For drugs that CLSI does not have a reference bacteria and range, published information was adapted and experiment conducted the same way as their structural analogs (Dorey et al., 2017b; Smith, 2017).

### Checkerboard Assay

#### *In vitro* Synergism of FF + TAP Against *A. pleuropneumoniae* and *P. multocida*

The synergistic interaction between FF and TAP was determined by checkerboard assay technique (Hsieh et al., 1993). Prior to the addition of bacteria, two-fold serial dilutions of FF and TAP (range, 0.125 × MIC to 3 × MIC) were made to create different concentration combinations in each well. In cases where the serial dilutions exceeded the capacity of the 96 well-plate, a second plate was used to complete the checkerboard analyses. The bacteria at a final concentration of 5 × 10^5^ CFU/mL was added to each well. The FICI was calculated as the sum of the MIC of each compound used in combination, divided by the MIC of each compound used alone as the following:

FIC=(FF)(MICofFFincombinationwithTAP)/(MIC⁢of⁢FF⁢alone).

FIC=(TAP)(MICofTAPincombinationwithFF)/(MIC⁢of⁢TAP⁢alone)

(1) FICI=FIC+(FF)FIC(TAP)

The results were interpreted using the following criteria: synergism (FICI ≤ 0.5), no interaction (0.5 < FICI < 4) and antagonism (FICI ≥ 4) (Odds, 2003).

#### *In vitro* Synergism of FF and Other Antimicrobial Agents

*P. multocida* isolates in which the FF + TAP in combination showed synergism were then exposed to the combinations of FF and the other two classes of antimicrobial agents, namely tetracyclines (OTC and DOX) and macrolides (ERY and TYL) using checkerboard techniques as described above.

### Time-Kill Assay

This study was conducted according to the standard protocol (The National Committee for Clinical Laboratory Standards [NCCLS], 1999) with some modifications. Cultures undergoing exponential growth were diluted to 5 × 10^6^ CFU/mL in BHI (Difco laboratories, Detroit, MI, United States). All *A. pleuropneumoniae* strains were diluted into BHI supplemented with 5 μg/mL NAD. Tubes containing 5 mL cultures were exposed to either drug alone (FF or TAP at a concentration equal to 0.5 × MIC) or an FF + TAP combination (at a concentration of 0.5 × MIC of either drug) and incubated at 37°C with shaking. For *A. pleuropneumoniae*, the environment contained 5% CO_2_. Note that the concentration of each drug in the combination was also tested at 1 × MIC as in the previous publications (Wei et al., 2016a, b). A tube containing 5 mL of culture without antimicrobial agents was used as control group. Aliquots of 20 μL obtained from each cultured tube were inoculated on chocolate agar for colony counts after 10-fold serial dilution at 0, 2, 4, 8, 12, and 24 h post-inoculation. Synergy was defined as a ≥2 log_10_ reduction in CFU/mL of the drugs in combination compared to the most active single drug after 24 h (The National Committee for Clinical Laboratory Standards [NCCLS], 1999; EUCAST, 2000).

### Multistep Resistance Studies

Multistep resistance studies were conducted with two clinical isolates of *A. pleuropneumoniae* that exhibited synergistic MIC values of FF 0.125 μg/mL + TAP 0.5 μg/mL (isolate No. 4 in Table 2) and FF 1 μg/mL + TAP 2 μg/mL (isolate No. 30 in Table 2), respectively. BHI solution supplemented with 5 μg/mL NAD was combined with FF or TAP or FF + TAP at one eighth of the synergistic concentration for each respective isolate. Mixtures were inoculated with cultures of each respective isolate of *A. pleuropneumoniae* at exponential-phase at a final concentration of ∼1 × 10^6^ CFU/mL. The cultures were incubated at 37^*o*^C for 24 h in a 5% CO_2_ environment and subcultured daily in fresh medium with antibiotics daily for 12 days. The MIC was evaluated after every third passage using the microdilution method as described above. Any increase in MIC ≥4 folds relative to the initial value was defined as the acquisition of resistance (Drago et al., 2005a, b).

**TABLE 1:** Distribution of minimum inhibitory concentration (MIC) value for FF and TAP tested against *A. pleuropneumoniae* (*n* = 58) and *P. multocida* (*n* = 79) isolated from swine.

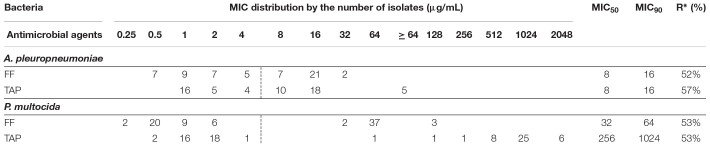

*FF, florfenicol; TAP, thiamphenicol; MIC, minimum inhibitory concentration. ^∗^R (%), resistance percentage. Resistance breakpoint of FF against A. pleuropneumoniae and P. multocida from swine (the dotted line) refers to Clinical and Laboratory Standards Institute [CLSI] (2018) Performance Standards for Antimicrobial Disk and Dilution Susceptibility Tests for Bacteria Isolated from Animal (Vet 08). Resistance breakpoint of TAP against A. pleuropneumoniae and P. multocida from swine (the dotted line) is derived from bimodal distribution data in this study and previous publications (Inamoto et al., 1994; Yoshimura et al., 2002; Morioka et al., 2008). MIC_50_ = antibacterial drug concentration that inhibit 50% of the bacterial population. MIC_90_ = antibacterial drug concentration that inhibit 90% of the bacterial population.*

**TABLE 2:** *In vitro* inhibitory activity of FF and TAP alone and in combination against *A. pleuropneumoniae* by checkerboard assay.

**Isolates (serovars)**	**MIC of each drug (μg/mL) when used alone**		**MIC of each drug (μg/mL) when used in combination**		**Fold MIC reduction**		**FICI**
	**FF**	**TAP**	**FF**	**TAP**	**FF**	**TAP**	
**Sensitive Strains (MIC of FF ≤ 2 μg/mL)**							
1 (1)	1	8	0.0625	1	1/16	1/8	0.1875
2 (1)	2	4	0.5	1	1/4	1/4	0.5
3 (1)	2	4	0.125	2	1/16	1/2	0.5625
4 (5)	1	1	0.125	0.5	1/8	1/2	0.625
5 (15)	0.5	1	0.0625	1	1/8	1/2	0.625
6 (5)	1	1	0.25	0.5	1/4	1/2	0.75
7 (5)	0.5	1	0.125	0.5	1/4	1/2	0.75
8 (5)	0.5	1	0.125	0.5	1/4	1/2	0.75
9 (5)	0.5	1	0.125	0.5	1/4	1/2	0.75
10 (2)	2	1	0.5	0.5	1/4	1/2	0.75
11 (1)	2	1	0.125	0.5	1/4	1/2	0.75
12(5)	1	1	0.25	0.5	1/4	1/2	0.75
13 (1)	2	2	0.5	1	1/4	1/2	0.75
14 (15)	0.5	1	0.125	0.5	1/4	1/2	0.75
15 (2)	0.5	1	0.125	0.5	1/4	1/2	0.75
16 (1)	1	8	2	4	1/2	1/2	1
17 (1)	1	4	2	2	1	1/2	1
18 (1)	2	1	1	0.5	1/2	1/2	1
19 (5)	1	1	0.5	1	1/2	1	1
20 (1)	2	2	1	1	1/2	1/2	1
21 (15)	0.5	1	0.25	0.5	1/2	1/2	1
22 (15)	1	16	1	2	1	1/8	1
23 (1)	1	1	1	1	1	1	2
**Intermediate Strains (MIC of FF = 4 μg/mL)**							
24 (1)	4	1	0.5	0.5	1/8	1/2	0.625
25 (1)	4	4	0.5	2	1/8	1/2	0.625
26 (1)	4	2	1	1	1/4	1/2	0.75
27 (1)	4	2	1	1	1/4	1/2	0.75
28 (1)	4	2	1	1	1/4	1/2	0.75
**Resistant Strains (MIC of FF ≥ 8 μg/mL)**							
29 (1)	16	16	1	2	1/16	1/8	0.1875
30 (1)	16	16	1	2	1/16	1/8	0.1875
31 (1)	16	16	2	2	1/8	1/8	0.25
32 (1)	16	16	2	2	1/8	1/8	0.25
33 (1)	8	8	1	2	1/8	1/4	0.375
34 (1)	16	16	4	4	1/4	1/4	0.5
35 (1)	16	16	4	4	1/4	1/4	0.5
36 (1)	8	16	2	4	1/4	1/4	0.5
37 (5)	32	≥ 64	16	4	1/2	≤ 1/16	≤0.5625
38 (1)	32	≥ 64	16	4	1/2	≤ /16	≤0.5625
39 (15)	16	≥ 64	8	4	1/2	≤ 1/16	≤0.5625
40 (1)	16	8	2	2	1/8	1/2	0.625
41 (5)	8	≥ 64	4	16	1/2	≤ 1/4	≤0.75
42 (5)	16	8	4	4	1/4	1/2	0.75
43 (1)	8	8	2	4	1/4	1/2	0.75
44 (1)	8	8	2	4	1/4	1/2	0.75
45 (5)	8	16	4	4	1/2	1/4	0.75
46 (5)	16	≥ 64	8	32	1/2	≤ 1/2	≤1
47 (1)	16	16	8	8	1/2	1/2	1
48 (2)	16	8	8	8	1/2	1	1
49 (7)	8	16	4	8	1/2	1/2	1
50 (1)	16	16	8	8	1/2	1/2	1
51 (1)	16	16	8	8	1/2	1/2	1
52 (1)	16	16	8	8	1/2	1/2	1
53 (1)	16	16	8	8	1/2	1/2	1
54 (1)	16	16	4	8	1/4	1/2	1
55 (5)	16	8	8	4	1/2	1/2	1
56 (1)	16	16	8	8	1/2	1/2	1
57 (1)	16	16	8	8	1/2	1/2	1
58 (1)	16	8	8	4	1/2	1/2	1

### Bacterial Challenge Study in Pigs

#### Experimental Design

Ten crossbred pigs (6 weeks old, 5 males and 5 females) were purchased from a pig farm in Nantou Country which was free from porcine reproductive and respiratory syndrome virus (PRRSV), porcine circovirus type 2 (PCV2) and classic swine fever virus (CSFV). *P. multocida*, serovar A (isolate No. 1 in Table 3) was used for the challenge. The pigs were divided randomly into five pairs (see below) and reared in separate pens for 5 days to allow time for environmental acclimation. All pigs were fed non-medicated feeds *ad libitum* and had free access to water. The bacterial inoculum was prepared from an overnight culture of *P. multocida* and reconstituted in phosphate-buffered saline (PBS) to 5 × 10^8^ CFU/mL (Oliveira Filho et al., 2015). Each pig was inoculated via intratracheal administration of *P. multocida* (1 mL) 30 min after drug treatments. The experiment was carried out for 5 days with the following treatments: Group 1 (negative control) intramuscular (IM) injections of 25% 2-pyrrolidone (0.1 mL/kg in PBS) on days 1, 3, and 5 (q 48 h). Group 2: daily IM injections with FF 2 mg/kg + TAP 4 mg/kg (q 24 h). Group 3: IM injections of FF 5 mg/kg + TAP 10 mg/kg on days 1, 3, and 5 (q 48 h). Group 4 (positive control 1) and Group 5 (positive control 2) were given recommended doses of FF at either 5 mg/kg, q 24 h (Group 4) or 15 mg/kg, q 48 h on days 1, 3, and 5 (Group 5) (Liu et al., 2015). After the 5th day of treatment, all pigs were sacrificed by electrocution and examined for any gross pathology and histopathology as well as bacterial re-isolation. The animal study was approved by the Institutional Animal Care and Use Committee of National Chung Hsing University (IACUC approval No. 105-079).

**TABLE 3:** *In vitro* inhibitory activity of FF and TAP alone and in combination against *P. multocida* by checkerboard assay.

**Isolates (serovars)**	**MIC of each drug (μg/mL) when used alone**		**MIC of each drug (μg/mL) when used in combination**		**Fold MIC reduction**		**FICI**
	**FF**	**TAP**	**FF**	**TAP**	**FF**	**TAP**	
**Sensitive Strains (MIC of FF ≤ 2 μg/mL)**							
1 (A)	0.5	1	0.0625	0.25	1/8	1/4	0.38
2 (D)	1	2	0.125	0.5	1/8	1/4	0.38
3 (A)	2	2	0.25	0.5	1/8	1/4	0.38
4 (A)	0.5	1	0.125	0.25	1/4	1/4	0.5
5 (A)	0.5	1	0.125	0.25	1/4	1/4	0.5
6 (A)	0.5	1	0.125	0.25	1/4	1/4	0.5
7 (D)	0.5	1	0.125	0.25	1/4	1/4	0.5
8 (D)	0.5	1	0.125	0.25	1/4	1/4	0.5
9 (D)	0.5	1	0.125	0.5	1/4	1/4	0.5
10 (A)	0.25	1	0.0625	0.5	1/4	1/4	0.5
11 (D)	0.5	2	0.125	0.5	1/4	1/4	0.5
12 (A)	0.5	2	0.125	0.5	1/4	1/4	0.5
13 (A)	1	2	0.25	0.5	1/4	1/4	0.5
14 (A)	1	2	0.25	0.5	1/4	1/4	0.5
15 (D)	1	2	0.25	0.5	1/4	1/4	0.5
16 (A)	2	4	0.5	1	1/4	1/4	0.5
17 (A)	0.5	2	0.125	0.5	1/4	1/4	0.5
18 (D)	2	2	0.5	0.5	1/4	1/4	0.5
19 (D)	1	2	0.0313	1	1/32	1/2	0.53
20 (A)	1	0.5	0.0313	1	1/32	1/2	0.53
21 (A)	0.5	1	0.0313	0.5	1/32	1/2	0.53
22 (A)	0.5	2	0.0313	1	1/16	1/2	0.56
23 (A)	2	2	0.125	1	1/16	1/2	0.56
24 (A)	2	2	0.25	1	1/8	1/2	0.63
25 (A)	0.5	1	0.125	0.5	1/4	1/2	0.75
26 (A)	0.5	1	0.125	0.5	1/4	1/2	0.75
27 (A)	0.5	0.5	0.125	0.25	1/4	1/2	0.75
28 (D)	0.5	1	0.125	0.5	1/4	1/2	0.75
29 (D)	0.5	1	0.125	0.5	1/4	1/2	0.75
30 (D)	0.5	1	0.125	0.5	1/4	1/2	0.75
31 (A)	0.5	1	0.125	0.5	1/4	1/2	0.75
32 (A)	2	2	1	0.5	1/2	1/4	0.75
33 (A)	0.25	2	0.0625	1	1/4	1/2	0.75
34 (A)	1	2	0.25	1	1/4	1/2	0.75
35 (D)	1	2	0.5	1	1/2	1/2	1
36 (A)	1	2	0.5	1	1/2	1/2	1
37 (A)	0.5	1	0.25	0.5	1/2	1/2	1
**Resistant Strains (MIC of FF ≥ 8 μg/mL)**							
38 (A)	128	2048	32	512	1/4	1/4	0.5
39 (D)	64	2048	32	512	1/2	1/4	0.75
40 (A)	64	1024	16	512	1/4	1/2	0.75
41 (D)	64	2048	16	1024	1/4	1/2	0.75
42 (D)	128	1024	32	512	1/4	1/2	0.75
43 (D)	128	1024	64	256	1/2	1/4	0.75
44 (A)	64	2048	16	1024	1/4	1/2	0.75
45 (A)	64	512	32	256	1/2	1/2	1
46 (D)	64	1024	32	512	1/2	1/2	1
47 (D)	64	1024	32	512	1/2	1/2	1
48 (D)	64	1024	32	512	1/2	1/2	1
49 (D)	64	1024	32	512	1/2	1/2	1
50 (D)	64	1024	32	512	1/2	1/2	1
51 (D)	64	1024	32	512	1/2	1/2	1
52 (D)	64	1024	32	512	1/2	1/2	1
53 (D)	64	1024	32	512	1/2	1/2	1
54 (A)	64	1024	32	512	1/2	1/2	1
55 (A)	64	1024	32	512	1/2	1/2	1
56 (D)	32	1024	16	512	1/2	1/2	1
57 (D)	64	1024	32	512	1/2	1/2	1
58 (D)	64	1024	32	512	1/2	1/2	1
59 (D)	64	1024	32	512	1/2	1/2	1
60 (A)	64	512	32	256	1/2	1/2	1
61 (A)	64	1024	32	512	1/2	1/2	1
62 (D)	64	512	32	256	1/2	1/2	1
63 (D)	64	512	32	256	1/2	1/2	1
64 (D)	64	512	32	256	1/2	1/2	1
65 (A)	64	64	32	32	1/2	1/2	1
66 (D)	64	1024	32	512	1/2	1/2	1
67 (D)	64	512	32	512	1/2	1/2	1
68 (A)	64	256	32	256	1/2	1/2	1
69 (A)	64	512	32	512	1/2	1/2	1
70 (D)	64	512	32	256	1/2	1/2	1
71 (A)	64	1024	32	512	1/2	1/2	1
72 (D)	64	1024	32	512	1/2	1/2	1
73 (A)	64	128	32	64	1/2	1/2	1
74 (D)	64	1024	32	512	1/2	1/2	1
75 (D)	64	1024	32	512	1/2	1/2	1
76 (D)	64	1024	32	512	1/2	1/2	1
77 (D)	64	1024	32	512	1/2	1/2	1
78 (D)	32	2048	8	1024	1/2	1/2	1
79 (D)	64	2048	32	1024	1/2	1/2	1

#### Clinical Evaluation

Clinical evaluations including body temperature, body weight, appetite and clinical signs (behavior and respiratory signs) were recorded once daily for each pig. A pig was considered to have fever if its body temperature was higher or equal to 39.5°C. Respiratory signs including coughing, sneezing and dyspnea (Stipkovits et al., 2001; Brockmeier et al., 2002) were recorded. Each individual was scored using the following criteria: 0 (no signs), 1 (moderate), or 2 (severe). For the behavioral observations, individual’s scores were recorded as 0 (normal), 1 (less active), or 2 (depression). Rectal temperatures and body weight were recorded daily before feeding and cleaning the stys. Three days before challenge and 5 days post-challenge, blood samples were collected into 0.5 mL tubes containing potassium EDTA and hematological values were determined using IDEXX ProCyte DxTM (IDEXX, Westbrook, ME, United States). Samples for biochemical analysis were collected in 1 mL serum collection tubes containing heparin, and analyzed for total protein, albumin, and globulin using a Roche Hitachi 717 Chemistry Analyzer (Hitachi, United States).

#### Gross and Histopathological Examinations and Bacterial Re-isolation

Complete necropsies were performed on all pigs. Macroscopic lung lesions of each lobe was assigned a score following the method used by Halbur et al. (1996) and the total percentage of lung area with pneumonia was estimated blindly by three veterinarians to get a mean score.

For histopathological examination, tissues from all lung lobes, tracheal, hilar lymph node, tonsil, liver, kidney, and inguinal lymph node were collected, processed and interpreted by veterinary pathologist from the Animal Disease Diagnosis Center (ADDC) at National Chung Hsing University (NCHU). Different parts of lung tissue including apical and cardiac parts of cranial lobes from both sides of the lung and hilar lymph nodes were examined for the presence of pathogenic bacteria.

## Results

### Resistance Profiles

The FF MIC for *A. pleuropneumoniae* reference strain using BHI/NAD was 0.5 μg/mL and falls within the acceptable range of 0.25–1 μg/mL (Clinical and Laboratory Standards Institute [CLSI], 2018). Results of the *in vitro* susceptibilities of *A. pleuropneumoniae* and *P. multocida* isolates to FF and TAP are summarized in Table 1 with values presented as the MIC distributions, MIC_50_, MIC_90_ and % resistance values. *In vitro* susceptibility testing revealed that FF was more effective than TAP overall against isolates of both *A. pleuropneumoniae* and *P. multocida.*

### Checkerboard Assay

#### *In vitro* Synergism by FF + TAP Combination

**FIGURE 1:**
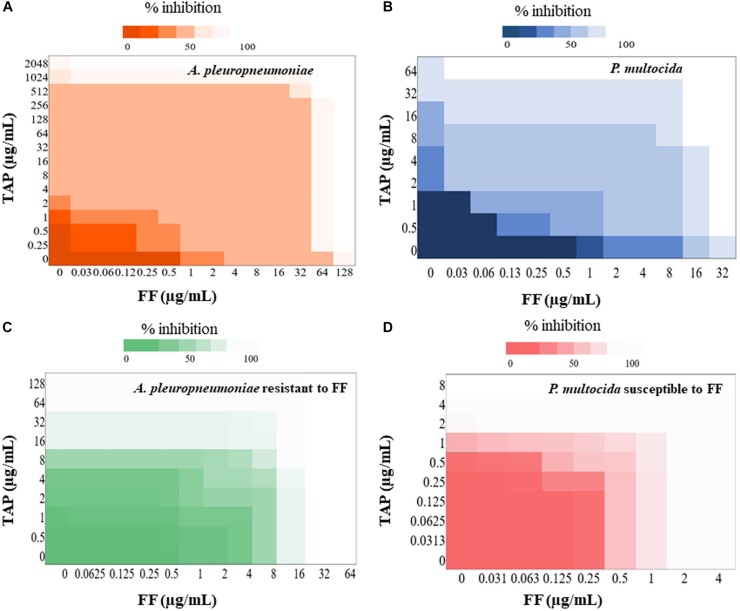
Heat plot showing synergy of FF and TAP. Checkerboard analyses showing the percentage inhibition on the combined effect of FF and TAP against **(A)** *A. pleuropneumoniae* **(B)** *P. multocida* **(C)** *A. pleuropneumoniae* resistant to FF and **(D)** *P. multocida* susceptible to FF.

The FF + TAP combination showed synergy (FICI ≤ 0.5) on 17% of the *A. pleuropneumoniae* isolates and 24% of *P. multocida* isolates (Tables 2, 3). The percentages increased to 33% (19/58) against *A. pleuropneumoniae* isolates and 32% (25/79) against *P. multocida* isolates if FICI ≤ 0.625 was considered. All isolates of *A. pleuropneumoniae* in which FF + TAP exhibited synergism belonged to serovar 1 (Table 2). Figures 1A,B provide heat map plots for the percentage of inhibition of both pathogenic bacterial species.

If isolates are classified as susceptible or resistant based upon the MIC breakpoint for FF, 40% (12/30) of resistant isolates of *A. pleuropneumoniae* showed synergism or FICI ≤ 0.625 whereas only 22% (5/23) of susceptible isolates exhibited the same. By comparison, synergism and FICI ≤ 0.625 was observed in 65% (24/37) of susceptible *P. multocida*, isolates as opposed to only 2.4% (1/42) of resistant isolates (Figures 1C,D).

### *In vitro* Synergism Between FF and Other Antimicrobial Agents

The *in vitro* antibacterial efficacies of FF + OTC, FF + DOX, FF + ERY, and FF + TYL combinations are summarized in Table 4. The results reveal that synergistic interactions (FICI ≤ 0.5) were observed in 40% (6/15) of the tested isolates for FF + OTC, 15% (2/13) for FF + DOX, 31% (5/16) for FF + ERY, and 13% (1/8) for FF + TYL. Consideration of synergism plus FICI ≤ 0.625 produced higher overall percentages for FF + OTC (7/15 or 47%), FF + DOX (9/13 or 69%) and FF + ERY (9/16 or 56%) combinations.

**TABLE 4:** *In vitro* inhibitory activity of FF with other antibiotic combinations against *P. multocida* by checkerboard assay.

**Bacterial isolates**	**FF + TAP**		**FF + OTC**		**FF + DOX**		**FF + ERY**		**FF + TYL**	
	**FICI**	**Activity**	**FICI**	**Activity**	**FICI**	**Activity**	**FICI**	**Activity**	**FICI**	**Activity**
2	0.38	S	0.75	N	0.63	N	0.50	S	1	N
3	0.38	S	0.75	N	0.63	N	0.50	S	-	-
4	0.50	S	0.50	S	0.63	N	0.63	N	0.75	N
5	0.50	S	0.50	S	0.75	N	0.75	N	0.50	S
6	0.50	S	0.75	N	–	–	1.50	N	1	N
7	0.50	S	0.75	N	0.75	N	1	N	1	N
8	0.50	S	0.75	N	0.63	N	0.75	N	–	–
9	0.50	S	–	–	0.75	N	0.75	N	–	–
10	0.50	S	0.56	N	0.38	S	0.50	S	–	–
11	0.50	S	0.38	S	0.75	N	0.75	N	–	–
12	0.50	S	0.50	S	0.50	S	0.50	S	–	–
13	0.50	S	0.75	N	0.56	N	0.63	N	–	–
15	0.50	S	0.50	S	–	–	0.63	N	–	–
16	0.50	S	1	N	0.63	N	0.50	S	1	N
17	0.50	S	1	N	–	–	0.75	N	1	N
18	0.50	S	0.50	S	0.63	N	0.63	N	0.75	N

**FICI, fractional inhibitory concentration index; S, synergism; N, no interaction; A, antagonism; FF, florfenicol; TAP, thiamphenicol; OTC, oxytetracycline; DOX, doxycycline; ERY, erythromycin; TYL, tylosin.**

### Time-Kill Assay

**FIGURE 2:**
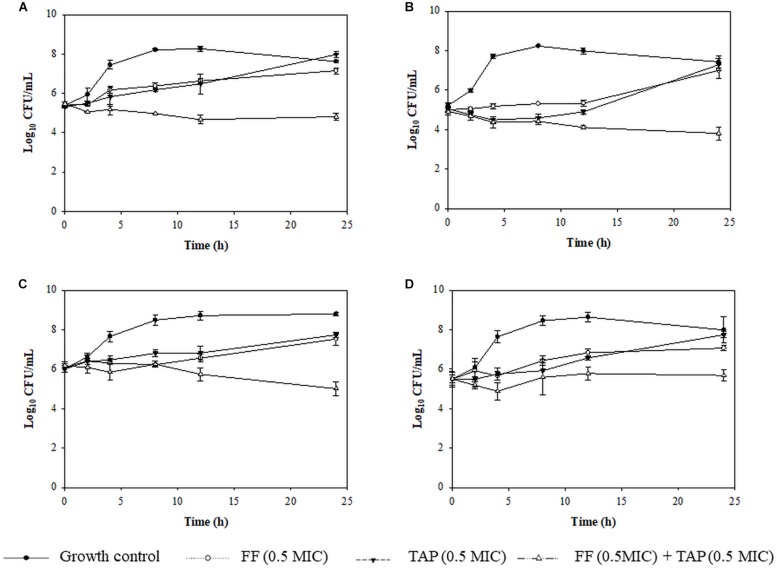
Time-kill assay showing the killing kinetic of FF, TAP alone and in combination against 4 isolates of intermediate **(B)** or resistant **(A,C,D)** *A. pleuropneumoniae* to FF **(A)** No.30 (FICI = 0.1875), **(B)** No. 25 (FICI = 0.625), **(C)** No. 42 (FICI = 0.75), and **(D)** No. 50 (FICI = 1) which served as the negative control. Refer to Table 2 for strain details. Results show the mean ± the standard error of the mean (SEM) from three independent experiments.

**FIGURE 3:**
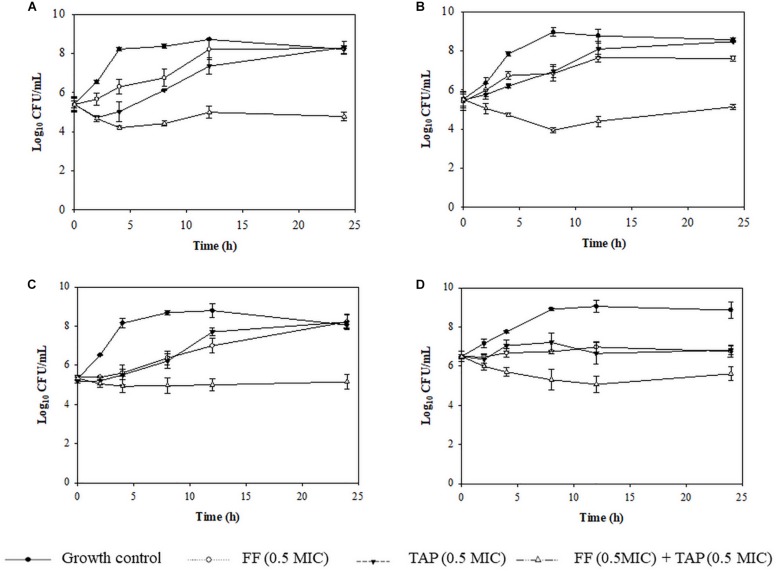
Time-kill assay showing the killing kinetics of FF, TAP alone and in combination against 4 isolates of susceptible *P. multocida* **(A)** No. 1 (FICI = 0.38), **(B)** No. 21 (FICI = 0.53), **(C)** No. 25 (FICI = 0.75), and **(D)** No.36 (FICI = 1) which serves as the negative control. Refer to Table 3 for strain details. Results show the mean ± the standard error of the mean (SEM) from three independent experiments.

Time kill assays were performed to verify evidence for synergism or FICI ≤ 0.625 and one FICI ≤ 0.75 in the checkerboard assay. The results for representative *A. pleuropneumoniae* isolates and *P. multocida* isolates are shown in Figures 2, 3, respectively. All three time kill assays with FF + TAP combinations at reduced dosage confirmed the synergism against intermediate and resistant *A. pleuropneumoniae* (Figures 2A–C), susceptible *P. multocida* (Figures 3A–C) which produced more than 2 log_10_ CFU/mL reductions compared to the largest reductions by FF or TAP alone at 24 h.

### Assessment of Resistance Induction at Sub-Inhibitory Concentrations

**FIGURE 4:**
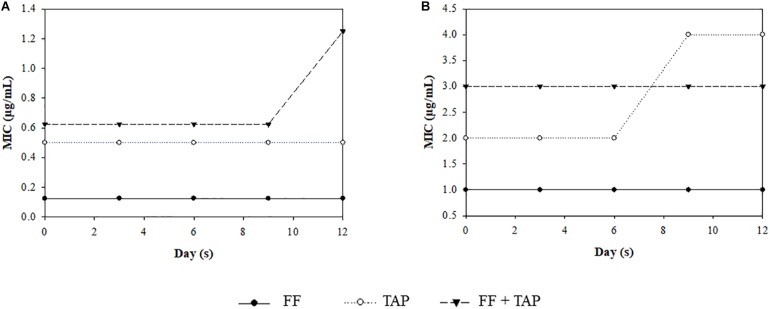
MIC change after serial exposure of *A. pleuropneumoniae* **(A)** strain No. 4, **(B)** strain No. 30, to sub-MIC level of FF, TAP, and FF + TAP in combination. Refer to Table 2 for strain information

After 12 passages, there was no apparent development of resistance with the only changes noted being a one-fold increase in MIC for FF + TAP group in the susceptible *A. pleuropneumoniae* strain and a one-fold MIC increase for the TAP group in the resistant *A. pleuropneumoniae* strain after the 9^*th*^ passage. The FF + TAP combination did not show any increased frequency for inducing *A. pleuropneumoniae* resistance compared to either drug when used alone (Figure 4).

### Bacterial Challenge Study in Pigs

#### Clinical Signs

After challenge, all pigs developed a low-grade fever on day 1. The medicated treatment groups (Groups 3, 4, and 5) recovered 1–2 days faster than the negative control group (Group 1), which developed a moderate to severe respiratory distress at day 2 post-inoculation that lasted until the time of euthanasia. Pigs in Group 2 also had mild respiratory distress at day 2 but were fully recovered by day 5, while the pigs in Groups 3, 4, and 5 did not show any respiratory distress. Pigs in the negative control group exhibited decreased spontaneous activity and food intake while pigs in the other groups exhibited normal behaviors and appetite. There was no significant difference in the body weight gain (∼2 kg/5 days) among the five groups.

#### Gross and Histological Examination

**FIGURE 5:**
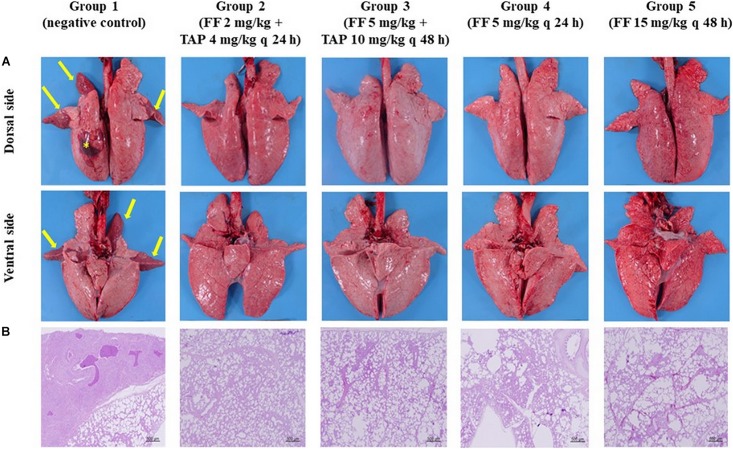
Gross lesions and histopathology of the pig lungs **(A)** Gross lesions of the pig lungs (dorsal and ventral side) in groups 1–5: Group 1 showed firm and dark red of the apical, cardiac parts of left cranial lobe and right cranial lobes (arrows) and 4 × 4 cm hematoma on dorsal surface of left diaphragmatic lobe (asterisks); Groups 2–5 showed few gross pathological signs. **(B)** Histopathology of the lung tissues (the right cranial lobe): Group 1 showed signs of interstitial pneumonia; The lung tissues from pigs in groups 2–5 showed only minor pathological changes (HE stain, 40×).

Figure 5 depicts the gross and histological features of lung tissues from each group. Most of the pigs showed no or only mild lung lesions (≥2% of the lung volume). However, pigs in the negative control group showed severe bronchopneumonia with moderate interstitial pneumonia (39.2% of the lung volume); apical and cardiac regions of the left cranial lobe, and the right cranial lobes were firm and dark red suggesting the pneumonia progressed from congestion phase to red hepatization phase. Under higher powered magnification (Supplementary Figure S2), inflammation was observed in the bronchial submucosa of negative controls, including the presence of lymphocytes and plasma cells. Alveoli were infiltrated by neutrophils, lymphocytes, macrophages and cellular debris. Besides, the alveoli of cardiac parts of the left and right cranial lobes lost their normal structures. The tonsils showed mild erosion and lymphoid depletion. Lung tissues from pigs in the Groups 2–5 showed only minor histopathological changes including multi-focal moderate interstitial pneumonia in each lobe of the lung and mild erosion of the tonsils.

#### Bacterial Re-isolation and Identification

*P. multocida* and *S. suis* were isolated from both sides of the cranial lobes of the lung from one pig in the negative control group. In contrast, no pathogenic bacteria were detected from pigs in all other treatment groups.

#### Hematology and Plasma Biochemistry Analyses

Comparisons of hematological profiles (Supplementary Table S2) and protein profiles (Supplementary Table S3) revealed no statistical differences (*P* > 0.5) among the five treatment groups with all values falling within or slightly above the normal range. Five days after bacterial challenge, the total white blood cell and neutrophil counts were mostly lower compared to the negative control group, while the lymphocyte, monocyte, eosinophil, and basophil counts were increased.

## Discussion

Porcine respiratory disease associated with *A. pleuropneumoniae* or *P. multocida* infection has increasingly impacted the health and industrial production of pigs (VanderWaal and Deen, 2018). As an alternative effort that focus on the identification of new antimicrobial agents for treating these disorders, the current study was undertaken to investigate the efficacy of combination treatment with existing antimicrobial agents FF + TAP that have previously shown evidence of efficacy against various pathogenic bacteria, including *P. multocida* in pigs (Wei et al., 2016b).

In the present study, results from *in vitro* antimicrobial susceptibility testing serve to expand and provide greater evidence for the efficacy of FF and TAP against porcine *A. pleuropneumoniae* and *P. multocida* in Taiwan. The resistant breakpoint of FF MIC against *A. pleuropneumoniae* and *P. multocida* were both at 8 μg/mL (Clinical and Laboratory Standards Institute [CLSI], 2018). Although the MIC breakpoint for TAP against these two pathogens in swine has not been established by the CLSI, it is defined as 8 μg/mL on the basis of bimodal distribution data in our study (Supplementary Figure S1) and previous publications (Inamoto et al., 1994; Yoshimura et al., 2002; Morioka et al., 2008). Consequently, 52% of the *A. pleuropneumoniae* isolates and 53% of the *P. multocida* isolates tested demonstrated FF resistance while 57 and 53% of respective bacteria were resistant to TAP. In comparison, studies from the United States and 11 European countries during year 2000–2006 showed almost 100% susceptibility to FF for both *A. pleuropneumoniae* and *P. multocida* (Priebe and Schwarz, 2003; Dayao et al., 2014; Jong et al., 2014; Sweeney et al., 2017), while 34% of *A. pleuropneumoniae* isolates were resistant to FF in Korea (Yoo et al., 2014). These data indicate that both respiratory pathogens in swine have developed stronger resistance to amphenicols in Taiwan, which highlights the need for more effective therapeutic alternatives or alternative strategies such as improved precision diagnosis, better and wider use of vaccines, probiotics and improved biosecurity (McEwen and Fedorka-Cray, 2002; Boehme et al., 2015).

Combination of antimicrobial agents is often presented as one of the few remaining effective strategies for the treatment of clinical diseases for which standard treatments have become ineffective. When 2 or more drugs are combined, the combinational effect can be defined as synergism (FICI ≤ 0.5), no interaction (0.5 < FICI < 4), or antagonism (FICI > 4) (Odds, 2003) based on microdilution checkerboard assay. Some researchers have further defined various levels of no interaction to be partial synergism (0.5 < FICI < 1), additivity (FICI = 1) and indifference (1 < FICI < 4) (Moody, 2004; Leu et al., 2014; Lee et al., 2017). While the reproducibility of checkerboard assay remains a concern for having distinct multiple fine levels of classification between synergism and antagonism, we chose to use the 3 levels as defined by Odds (2003). Nevertheless, due to the same reproducibility concern, it is also well practiced that for strains with FICI values slightly above 0.5, their synergism are further confirmed with more reliable methods such as time-kill assay (Bayer and Morrison, 1984; White et al., 1996). The combination of FF + TAP has been proven effective against *A. hydrophila*, *P. multocida*, *S. aureus*, *S. hyicus*, and *S. suis in vitro*, as well as in animal models against *A. hydrophila*, *S. aureus*, and *P. multocida* in fish, mouse and chicken, respectively (Wei et al., 2016a, b; Assane et al., 2019). The FF + TAP combination was also demonstrated effective against strains that were originally resistant (Wei et al., 2016a, b). In the current research, the study was extended to the synergistic combination against *A. pleuropneumoniae* and *P. multocida* in swine. The checkerboard assay initially showed synergism of the antibiotic combination against less than 25% of bacterial isolates; however, while this may seem low for a promising combination, it is possible that the two-fold dilutions used in the current design yielded a higher percentage of FICI index of 0.625, as suggested previously (Wei et al., 2016a, b). The isolates that were formerly designated as showing FICI ≤ 0.625 were later found to exhibit full synergism by time kill assay (Supplementary Table S1). In fact, the inclusion of isolates that met the criteria of ≤ 0.625 as cut-off value increased the percentage of synergism to 32% for both *A. pleuropneumoniae* and for *P. multocida.* Further analysis of susceptible and resistant strains (categorized by FF MIC breakpoint) revealed that the percentage of isolates displaying synergism or FICI ≤ 0.625 increased to 40% for resistant *A. pleuropneumoniae* isolates and 65% for susceptible *P. multocida*. It is interesting to note that the synergy against *A. pleuropneumoniae* was only observed in serovar 1, the predominant serovar of *A. pleuropneumoniae* in Taiwan (Yang et al., 2011; Liao et al., 2019), and 80% of which were FF-resistant strains (Table 2). However, the synergy against *P. multocida* did not show differences between serovars. Based on the above observations, it seems the presence of synergistic effects were strain-specific, as synergy was linked to species and resistance traits. Variability during synergy evaluation has already been reported by various authors (Tascini et al., 2013; Soren et al., 2015; Pollini et al., 2017), suggesting that the testing of individual different strains is required.

In order to ascertain whether isolates showing FICI ≤ 0.625 by the checkerboard assay could actually be synergistic by other common measures, the time-kill assay was performed as it has been demonstrated to reveal synergy more often than the checkerboard assay and it is reported to be more reliable in the prediction of *in vivo* synergism (Bayer and Morrison, 1984; Chadwick et al., 1986; White et al., 1996; Dong et al., 2017). Of all 9 *A. pleuropneumoniae* strains that show FICI ≤ 0.625, more than 78% (7/9) exhibits more than two log_10_ reduction in bacterial growth. For the 6 *P. multocida* isolates in which FICI ≤ 0.625, more than 83% (5/6) showed synergism (>2 log_10_ reduction) (Supplementary Table S1). These results clearly showed the importance of utilizing both methods in the evaluation of antimicrobial synergism. Accordingly, it is evidenced that isolates classified as FICI ≤ 0.625 in this study may actually be synergistic (FICI ≤ 0.5) such that the percentage synergism of *A. pleropneumoniae* and *P. multocida* isolates were actually higher than the current data indicates.

The mechanism by which the FF + TAP combination produces synergism, in particular the preferable activity against resistant *A. pleropneumoniae*, is not fully understood but may be related in part to the FF efflux pumps encoded by the floR gene detected in the FF resistant *A. pleropneumoniae* isolates (Kucerova et al., 2011; Yoo et al., 2014). Further investigations are required to address this possible relationship. Alteration of bacterial cell membrane permeability by FF to facilitate the bacterial uptake of TAP (or other antimicrobial agents) has been demonstrated as another possible mechanism (Wei et al., 2016b). Based on this theory, synergism between FF and other antibiotics (in addition to TAP) is also likely. In fact, the *in vitro* synergism between FF and other classes of antimicrobial agents against various bacteria including FF + amoxicillin against *Staphylococcus aureus*, *Escherichia coli*, *Proteus mirabilis* (Choi et al., 2011) and pathogenic bacteria of fish (Lee et al., 2010) and FF + OTC against *P. aeruginosa* (Wei et al., 2016b) have been reported. With respect to the same three antibiotic combinations evaluated in this study, Abu-Basha et al. (2012) demonstrated that 90.4% of the FF + OTC, 81% of the FF + ERY and 47.7% of the FF + DOX were synergistic or showing FICI ≤ 0.75 against 18 resistant *E. coli* isolates. In contrast, the combinations of FF with tulathromycin, ceftiofur, tilmicosin and enrofloxacin showed no evidence of synergism in all susceptible and intermediate strains (10 isolates) of bovine *P. multocida* (Sweeney et al., 2008). In the current study, more than 50% synergistic activity or FICI ≤ 0.75 of FF with OTC, DOX, and ERY (but not FF + TYL) were evident against *P. multocida* isolates that showed synergy to FF + TAP. The reasons why FF was synergistic with OTC, DOX, and ERY remain to be elucidated but it seems to be related to not only drug classes but also specific drugs, as FF is synergistic to ERY but not TYL. It is also apparent that the combination works better at specific combination ratios (see more discussion below on working ratio of TYL). Further study is warranted to investigate whether FF has the ability to act as a central modulator that facilitates synergistic effects with other antimicrobials (Wei et al., 2016b).

Exposure to antimicrobial concentrations that are sub-MIC play a vital role in the development of antimicrobial resistance (Andersson and Hughes, 2014; Ge et al., 2017). At reduced concentrations, any synergistic combination should be evaluated and shown to not increase the possibility of resistance development. Previous studies have indicated that increases in MIC ≥ 4 fold compared to the starting MIC value after five serial passages (Drago et al., 2005a, b) is evidence of resistance acquisition. In the current study, the MIC of FF + TAP increased by only one-fold in one susceptible and one resistant *A. pleuropneumoniea* strain over 12 passages, indicating it did not induce bacterial resistance after 12 passages. This is consistent with previously published data that the resistant mutation frequency is very low when *P. multocida* is grown with FF + TAP combinations (Wei et al., 2016b). Despite that phenotypic adaptation may be the probable cause of some of the observed MIC changes, as opposed to the development of true resistance (Hammer et al., 2012), these results provided evidence for the effectiveness and safety of the combinational therapy of FF + TAP compared to the use of FF or TAP in isolation.

The *in vivo* efficacy of FF + TAP was also tested in this study. Since *A. pleuropneumoniea* infections normally produce a clinical manifestation with high mortality rate, intra-tracheal inoculation of *P. multocida* was used as a clinical pneumonia model (Dowling et al., 2002; Oliveira Filho et al., 2018). While all parameters in the two treatment groups and the two positive control groups showed little or no clinical, histological or biochemical abnormalities after the bacterial challenge, the negative control group showed evidence of severe inflammation/infection. Bacterial re-isolation did not find any *P. multodica* in the treatment and positive control groups. Therefore, based on the overall assessment, it was concluded that FF + TAP combinations, whether in a single-dose daily or every other day regimen, were equally effective as the recommended doses of FF in eliminating the pathogens and preventing infection, which correlated well with the *in vitro* results. A larger scale field study is warranted in the future.

It is worth noting that the optimal ratios for drug combination dosages *in vivo* exhibited good proportionality to the optimal ratios with respect to *in vitro* MIC results. The MIC of FF could be reduced by up to 87.5% (1/8 MIC) for *P. multocida* when combined with 1/4 MIC of TAP, a 1:2 ratio. This ratio has been reported previously to be effective in both mice and chickens (Wei et al., 2016a, b). In contrast, FF + TYL combination at a ratio of 2:1 (FF 50 mg + tylosin tartrate 25 mg per mL; intramuscular administration) is available as a commercial product in Korea for pigs and dogs (Kim et al., 2008, 2011) based on broader antimicrobial spectrum. While the underlying rationale for each combination may differ, it seems plausible that an optimal ratio may exist for maximizing the synergistic efficacy of different drug combinations. The potential benefits of significant reductions in individual dosage of FF and TAP is evident in that synergistic FF + TAP could feasibly reduce the drug residues and thereby the withdrawal time. In particular, because FF at therapeutic doses is eliminated from the pig’s body by first-order kinetics with a terminal half-life of 11–17 h (Liu et al., 2002, 2003; Jiang et al., 2006; Dorey et al., 2017a), a dosage reduction would reduce both residue levels and the residual time. For instance, due to the greater reduction of FF in the combination treatment, a shorter withdrawal time is possible (18 days for FF in swine according to Veterinary Medicines Directorate [VMD], 2013). This is also supported by a previous investigation that reveals significantly lower tissue drug residues within periods as short as 1 day in most tissues of broiler chicken following FF + TAP administration (Rairat et al., 2019). It is further supported by Assane et al. (2019) recently who found that an FF + TAP combination with FICI ≤ 0.75 *in vitro* is also effective *in vivo* against *A. hydrophila* in Nile tilapia when 63% less antibiotic was used, resulting in a survival rate of 86%. If these results can be extended to pigs, there would be reduced risk of toxicity and an increase in human food safety.

## Conclusion

The present study demonstrated the potential benefits of using synergistic FF + TAP combination to combat *A. pleuropneuminiae* and *P. multocida* associated respiratory infections in pigs both *in vitro* and *in vivo*. These results were generally in agreement with two previous studies that were conducted against different bacterial species/isolates *in vitro* and in different animal species (Wei et al., 2016a, b; Assane et al., 2019). Different degrees of *in vitro* synergism between FF plus OTC or ERY against *P. multocida* were also evident. Finally, the potential of FF + TAP or FF in combination with other antibiotics as new drugs for the treatment of swine diseases warrant further investigation.

## Data Availability Statement

All datasets generated for this study are included in the manuscript/Supplementary Files.

## Ethics Statement

The animal study was reviewed and approved by the Institutional Animal Care and Use Committee of National Chung Hsing University (IACUC approval No. 105-079).

## Author Contributions

C-CC conceived the study and designed the experiments. PR conducted the *in vitro* experiments and analyzed the data. H-CK provided the bacterial isolates and identified their species and serovars. C-HS, T-LL, and S-YY carried out the *in vivo* efficacy study. PR, TR, C-HS, T-LL, and S-YY drafted the manuscript. C-CC and TV critically revised the manuscript. All authors read and approved the final manuscript.

## Conflict of Interest

The authors declare that the research was conducted in the absence of any commercial or financial relationships that could be construed as a potential conflict of interest.
