# Supplementary material for: In vitro and in vivo Synergistic Effects of Florfenicol and Thiamphenicol in Combination Against Swine Actinobacillus pleuropneumoniae and Pasteurella multocida
Source: Front Microbiol. 2019 Oct 30;10:2430. doi: 10.3389/fmicb.2019.02430 (PMC6842999; doi:10.3389/fmicb.2019.02430)
Supplement: Supplementary file 1 [file Data_Sheet_1.pdf]

Supplementary Material

In Vitro and *In Vivo* Synergistic Effects of Florfenicol and Thiamphenicol in Combination against Swine *Actinobacillus pleuropneumoniae* and *Pasteurella multocida*

Porjai Rattanapanadda[1], Hung-Chih Kuo[2], Thomas W. Vickroy[3], Chi-Hsuan Sung[1], Tirawat Rairat[1], Tsai-Lu Lin[1], Sze-Yu Yeh[1], and Chi-Chung Chou[1,*]

[1]Department of Veterinary Medicine, College of Veterinary Medicine, National Chung Hsing University, Taichung, Taiwan
[2]Department of Veterinary Medicine, College of Veterinary Medicine, National Chiayi University, Chiayi, Taiwan
[3]Department of Physiological Sciences, College of Veterinary Medicine, University of Florida, Gainesville, Florida, USA

Running head: Amphenicols Synergism Against *A. pleuropneumoniae* and *P. multocida*

Keywords: synergism, florfenicol, thiamphenicol, *Actinobacillus pleuropneumoniae*, *Pasteurella multocida*

***Correspondence:**
Chi-Chung Chou
Department/College of Veterinary Medicine,
National Chung Hsing University,
145 XingDa Rd. Taichung, Taiwan
Email: ccchou@nchu.edu.tw

1. Supplementary Tables

Table S1: Time kill synergy results within the same class of antibiotics florfenicol (1/2 MIC) and thiamphenicol (1/2 MIC) against *A. pleuropneumoniae* and *P. multocida* isolates FICI ≤ 0.625 at 24 hours.

Bacteria	Isolate number	FICI value	Difference in bacterial concentration (no. of $\log_{10}$ CFU/ml) between combination and most active single agent at 24 hours*
A. pleuropneumoniae	4	0.625	-3.19
	5	0.625	-3.32
	24	0.625	-2.63
	25	0.625	-2.47
	37	≤0.5625	-3.54
	38	≤0.5625	-3.54
	39	≤0.5625	-2.89
	3	0.5625	NA
	40	0.625	NA
P. multocida	20	0.53	-3.56
	21	0.53	-2.85
	22	0.56	-2.48
	23	0.56	-2.54
	24	0.625	-3.29
	19	0.53	NA

*Calculation from (number of $\log_{10}$ CFU per milliliter of growth culture with FF + TAP combination at 24 hours of incubation) – (number of $\log_{10}$ CFU per milliliter of growth culture with the most active antibiotic alone at 24 hours of incubation).

- Synergy was defined as a ≥ 2 log10 reduction in CFU/mL of the drugs in combination compared to the most active single drug after 24 hours.
- Negative values indicate a lower final colony count with the combination than with the most active single agent.
- NA denotes “can not revive”.

Table S2. The average hematological values and their reference intervals of the pigs in Groups 1-5 (G1-G5) before and after bacterial challenging

**Hematological parameters**	**Pre-challenging***					**Day 5 post-challenging***					**Reference intervals**
	**G1**	**G2**	**G3**	**G4**	**G5**	**G1**	**G2**	**G3**	**G4**	**G5**	
RBC (M/μL)	7.2	7.0	8.2	*9.6*	8.0	7.5	6.1	7.3	8.1	9.0	5.5 - 9.1
HCT (%)	35.5	39.8	39.7	*43.9*	39.7	37.6	34.0	34.5	36.7	*43.7*	28.3 - 42.7
HGB (g/dL)	10.5	11.4	11.9	12.7	11.8	11.1	10.1	10.8	10.9	*13.2*	8.8 - 12.7
MCV (fL)	49.0	56.5	48.5	45.9	49.7	50.1	55.6	47.5	45.1	49.1	38.4 - 59.3
MCH (pg)	14.5	16.1	14.5	13.3	14.8	14.8	16.4	14.8	13.4	14.9	11.1 - 18.4
MCHC (g/dL)	29.7	28.6	30.0	28.9	29.8	29.5	29.6	31.2	29.7	30.3	27.9 -32.4
RDW (%)	*35.8*	24.6	*33.5*	*34.2*	31.7	*35.6*	25.0	32.2	*33.4*	31.7	16.4 -32.3
WBC (K/μL)	*32.3*	*31.0*	*27.6*	23.2	*28.6*	*31.0*	*27.7*	24.2	*25.5*	22.4	5.4-25.2
NEU (K/μL)	*14.4*	12.3	*13.7*	8.3	11.9	10.4	10.8	9.5	8.6	6.3	0.8 - 13.4
LYM (K/μL)	*15.6*	*16.2*	12.6	13.6	14.6	*18.2*	14.5	12.8	*15.1*	14.3	3.8 - 14.9
MONO (K/μL)	*1.82*	*1.83*	1.15	0.96	1.62	*1.97*	*1.87*	1.61	1.17	1.24	0.22 - 1.71
EOS (K/μL)	0.35	*0.63*	0.09	0.24	0.47	*0.44*	*0.45*	0.22	*0.62*	0.52	0.05 - 0.40
BASO (K/μL)	0.02	0.04	0.02	0.02	0.02	0.06	0.04	0.05	0.04	0.04	0.01 - 0.15
PLT (K/μL)	603	722	626	180	*874*	593	667	519	723	*910*	208 - 873
MPV (fL)	12.0	9.4	11.3	10.9	10.8	12.2	9.5	12.1	12	10.9	No report

RBC, red blood cells; HTC, hematocrit; ; HGB, hemoglobin; MCV, mean corpuscular volume; MCH, mean corpuscular hemoglobin; MCHC, mean corpuscular hemoglobin concentration; RDW, erythrocyte distribution width; WBC, white blood cells; NEU, neutrophils; LYM, lymphocytes; MONO, monocytes; EOS, eosinophils; BASO, basophils; PLT, platelets; MPV, mean platelet volume; the italicized values are fall outside the respective reference interval; *the data between pre-challenging and day 5 post – challenging are not significantly different ($P > 0.05$).

Table S3. The average biochemistry values and their reference intervals of the pig in Groups 1-5 (G1-G5) before and after bacterial challenging

**Biochemistry parameters**	**Pre-challenging***					**Day 5 post-challenging***					**Reference intervals**
	**G1**	**G2**	**G3**	**G4**	**G5**	**G1**	**G2**	**G3**	**G4**	**G5**	
Total protein (g/dL)	*4.9*	*4.8*	5.8	*4.4*	*4.8*	*5.4*	*4.6*	*5.4*	*5.2*	6.6	5.8-8.5
Albumin (g/dL)	2.6	2.9	2.6	2.4	2.7	2.8	2.5	2.7	2.7	3.4	2.4-3.9
Globulin (g/dL)	*2.3*	*1.9*	*3.2*	*2*	*2.1*	*2.7*	*2.2*	*2.7*	*2.5*	*3.3*	3.4-4.5

*The data between pre-challenging and day 5 post – challenging are not significantly different ($P > 0.05$).

2. Supplementary Figures

(A) Distribution of MIC values of TAP against *A. pleuropneumoniae*

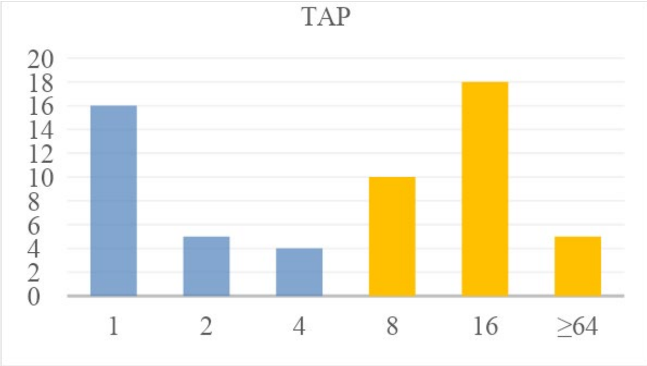

(B) Distribution of MIC values of TAP against *P. multocida*

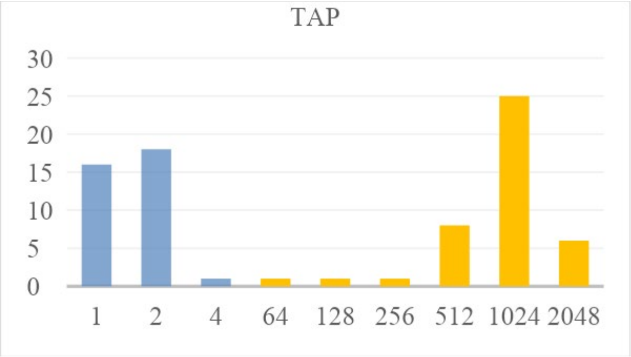

Figure S1. Bimodal distribution of MICs for TAP against (A) *A. pleuropneumoniae* (B) *P. multocida*

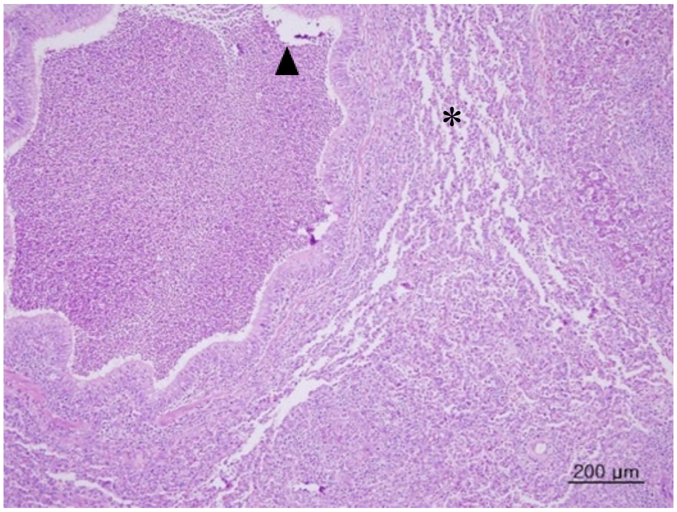

Figure S2. Histopathological features of the lung tissues in pigs challenged with *P. multocida* infection without any treatment in group 1; bronchial submucosa was infiltrated with lymphocytes and plasma cells (arrowhead), alveoli were infiltrated with inflammatory cells including polymorphonuclear cells, lymphocytes, macrophages and tissue debris (asterisks) (HE stain, 100X)
